# A Study on the Influence of Patient Participation on Patient Trust-Based on Sample Survey in China

**DOI:** 10.3389/fpsyg.2018.02189

**Published:** 2018-11-14

**Authors:** Tongwei Yang, Yijin Wu

**Affiliations:** ^1^School of Medicine, Shandong University, Jinan, China; ^2^School of Translation Studies, Center for Medical Humanities in the Developing World, Qufu Normal University, Qufu, China

**Keywords:** patient participation, the patient’s psychological contract, patient trust, shared decision making, China

## Abstract

Trust plays a central role in a doctor – patient relationship, and patient’s trust in doctors is the most important factor in trust relationship between the doctor and the patient. By reviewing the existing studies, this study proposes that patient participation is an antecedent variable influencing patient trust, and the patient can take the initiative to participate in diagnostic activities with the aim of establishing and maintaining the psychological contract with the doctor, thereby strengthening doctor-patient trust. In this study, we propose the model on the relationship between the patient participation, the patient’s psychological contract and the patient trust, and then conduct an empirical study. The research findings indicate that patient participation is an antecedent variable for the patient’s psychological contract and patient trust; the patient’s psychological contract is a mediator variable between the patient participation and the patient’s trust in doctor; patient participation can be categorized as three dimensions: information search, responsible behavior, and interpersonal relationship. Thus, we should encourage patient participation, advocate the shared decision-making, and promote the synergic improvement of interpersonal trust and universal trust between doctors and patients.

## Introduction

The relationship between doctors and patients is not only an important issue involved in reforming China’s healthcare, but also an issue in maintaining citizens’ health and constructing harmonious society. It has been shown that most medical disputes stem from the lack of the bond of trust between doctors and patients (Zheng, 2010a). Thus, rebuilding the broken bond of trust is one of important goals that the China’s state-owned hospitals are striving for (Li, 2010). It is clear that trust is central for the relationship between doctors and patients.

Trust can be defined as the practice that one party (trustor) is willing to rely on the actions of another party (trustee). Once the trustee is trusted, the power to dominate and control will be delivered to the trsutee (Li, 2005). Mainous et al. (2003) argued that trust between doctors and patients refers to the patient’s anticipation that the doctor will provide good medical service and maintain the patient’s interest. Drawing on the rational choice theory, Zheng (2010b) argued that the trust relationship between doctors and patients is characterized as the transfer of the patient’s right to the doctor, specifically, the patient autonomy is delivered to the doctor. Yang (2007) argued that trust refers to the positive anticipation of the vulnerable trustor for the trustee’s good performance. Based on the previous studies, this paper defines patient trust as the belief that patients think that doctors have the skills necessary for diagnosis and treatment, and will take patients’ interests into consideration first, thereby patients accepting the medical services in a reassuring manner.

The complexity of the medical service determines that patient trust is influenced by a good number of factors, among which the role of the patient participation can not be ignored. It is because health care is a very special profession where the patient should participate in the whole process of the medical service in person. The clinical decision making is deeply influenced by patient’s understanding and cooperation. Patient participation can promote mutual understanding, reduce the medical expenditure, improve the medical quality, and eventually enhance the patient’s trust in the doctor.

Since 1990s, the concept of the psychological contract has been widely applied to the marketing field. During transacting with the seller, besides focusing on the explicit articles such as the price, quality assurance and after-sales service, the client also has some potential expectations such as the salesman’s good attitude, quick and considerate after-sales service and dignity. Although these factors are hard to be quantified, standardized and specified, they are customary and should be incorporated into the client’s psychological contract (Blancero and Ellram, 1997). In recent years, the theory of the psychological contract has been introduced into the study of the relationship between doctors and patients. Zou (2010) argues that there is still a psychological contract between doctors and patients that refers to a series of subjective beliefs about mutual expectation existing between doctors and patients. Chen et al. (2013a) claims that the relationship between doctors and patients in essence is a kind of contractual relationship, and the patient’s psychological contract is a kind of subjectively and inherently psychological contract between doctors and patients, which is a combination of the emotional fitness and commitment fitness (Chen et al., 2013a).

Luo and Fan (2005) take the social service profession as the study object and find that there is a significant path relationship between the client’s psychological contract, client trust, client commitment, and client loyalty. Yu et al. (2011) demonstrates that both transaction psychological contract and relationship psychological contract have a significantly positive influence on the network trust, and the trust network also has a positive influence on the client’s repeat purchase practices. In the field of health care, the lack of the patient’s psychological contract is a decisive factor for triggering conflict between doctors and patients (Chen et al., 2013b).

**FIGURE 1:**
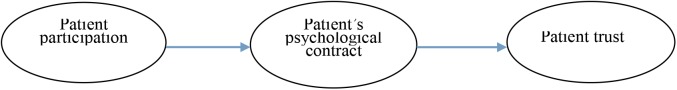
Conceptual model of this study.

The previous studies above indicates that: (1) the high-contact nature of the medical service warrants that the medical service not only needs doctor’s engagement, but also more patient participation and cooperation. Patient participation influences the mutual expectation and understanding between doctors and patients and the patient’s trust in the doctor; (2) the patient’s psychological contract includes the patient’ understanding of the responsibilities doctors and hospitals should assume and the responsibilities assumed by him/herself in the sense that it is a kind of psychological bond connecting the patient and the hospital. Thus, the research hypothesis is that the patient’s psychological contract serves as a mediator variable between the patient participation and the patient’s trust in doctor which can be shown in Figure 1.

## Materials and Methods

### Participants

In August, 2014 a total of 520 questionnaires were distributed to the inpatients and outpatients in Jining and Heze’s hospital of Shandong province. Finally, 487 valid questionnaires was obtained. The valid recovery rate of the questionnaire was 93.65%. The sample distribution is shown in the Table 1.

**Table 1:** Distribution of the survey samples.

Item	Category	Sample amount	Percent (%)
Sex	Male	232	47.64
	Female	255	52.36
Age	≤20 years’ old	28	5.75
	21–30 years’ old	164	33.68
	31–40 years’ old	99	20.33
	41–50 years’ old	61	12.53
	51–60 years’ old	63	12.94
	More than 60 years’old	72	14.78
Category of	Basic medical insurance	153	31.42
Medical	for urban employee		
insurance	New rural cooperative		
	medical system	203	41.68
	Urban citizens	112	23.00
	Other	19	3.90
Monthly income	Less than 3000 Yuan	259	53.18
	3000–5000 Yuan	180	36.96
	5000–8000 Yuan	34	6.98
	More than 8000 Yuan	14	2.87


### Measure

The Patient Participation Scale developed by Geng (2008) is used for measuring the patient participation. All the coefficients cronbach’s α of the three dimensions: responsible behavior, information search and interpersonal interaction in this scale are all greater than 0.7, with better internal consistency and higher reliability; meanwhile, the scale has good content validity and structural validity. The measurement articles are: (1) I have understood the information about the hospital or the doctor before visiting the doctor; (2) I have gained a basic understanding of my own symptoms and have had an expectation of how I will be treated before visiting the doctor; (3) I have searched for the relevant information and known what I should assist my doctor to do before a doctor’s visit; (4) I will try my best to describe my symptoms to the doctor during medical diagnosis or treatment; (5) I have completed medically necessary procedure as required during medical diagnosis or treatment; (6) I will cooperate fully with my doctor and thoroughly follow the requirements the doctor makes; (7) I will put forward new suggestions to improve the quality of the medical services; (8) I will take the initiative to communicate with my doctor; (9) I will do what I can do to help other patients if necessary.

“Client’s Psychological Contract Scale” developed by Luo (2006) is used to measure the patient’s psychological contract, and appropriate and necessary adjustment was made according to the medical service scenario and suggestions from the experts. The modified scale is as follows: (1) I believe that the hospital is willing to provide good medical services and facilities, and the doctor will consider my feelings sincerely; (2) I believe that the doctor will carefully weigh the treatment plan and save my medical expenses sincerely; (3) I believe that the hospital will optimize the care process and reduce my wait time; (4) I do not believe that my doctor will use expensive or inappropriate drugs or technologies to earn money; (5) I believe that the doctor is familiar with my physical condition, my hope, and requirement; (6) I believe that the doctor will explain to me patiently if I have some doubts about the therapeutic regimen or results; (7) I believe that the hospital will protect my interests and take the initiative to assume its own responsibility once any medical accident occurs; (8) I believe that the hospital will provide me with reliable and assuring quality services; (9) I believe that my doctor respects me sincerely instead of being perfunctory; (10) I believe that the hospital will make long-term quality and reputation assurance to its therapeutic results; (11) I believe that my treating doctor will care about my emotion, work and life sincerely; (12) I believe that my treating doctor attaches importance to his or her friendship with me.

The revised Chinese version of the Wake Forest Physician Trust Scale is used to measure the patient’s interpersonal trust in the doctor. This scale has good psychological properties and reliable validity (Dong and Bao, 2012), and its measuring articles include: (1) the doctor is capable of using the medical equipment skillfully when he or she is serving me; (2) the doctor will try his or her best to ensure my health; (3) the doctor always choose the therapy at his/her convenience rather than focusing on the therapy which is suitable for my status; (4) the doctor’s skills of don’t reach the level that I expect; (5) the doctor is very careful and considerate; (6) I feel that the therapeutic regimen that the doctor chooses is most suitable for me; (7) the doctor will explain the differences between all the possible therapeutic regimens that will be taken for me; (8) I feel that the doctor doesn’t listen carefully to my problem presentation; (9) the doctor puts my interests first, instead of his/her or hospital’s interests; (10) I can entrust the doctor with my life safety without any hesitation; (11) anyway, I trust my doctor.

### Statistical Analysis

Before conducting the statistical analysis, we test the reliability of the sample data. The reliability reflects the consistency or stability of the results obtained by the test tools, which is an index of the authenticity for the characteristics tested. Cronbach’s α coefficient refers to the consistency between the scores of various items in the scale, which belongs to a coefficient of internal consistency, reflecting whether it is consistent or not while all the participants are answering the questions. Guieford (1965) argued that if Crobach α < 0.35, it refers to the low reliability; if 0.35 ≤ Crobach α < 0.7 occurs, it is tolerable; if Crobach α ≥ 0.7, it refers to the high reliability. Nunnally (1978) argued that, in practice, Crobach α coefficient should be at least more than 0.5 and it is better if it is more than 0.7.

SPSS statistical software is used to analyze the reliability of the data obtained from the survey, and its results indicate that the internal consistency coefficients of three measure variables: the patient participation, the patient’s psychological contract and patient trust are 0.83, 0.96 and 0.81, all of which are more than 0.7. It can be argued that the consistency of each measuring item and the reliability of the data obtained from the measurement are satisfactory, and the measurement variables and the data obtained from the measurement can be used for further study and analysis. In what follows, Varimax rotation is then used to conduct factor analysis of the patient participation; According to the principle of structural equation model, the conceptual model constructed by us is then tested using AMOS 6.0 statistical analysis software.

## Results

### Factor Analysis of Patient Participation

Varimax rotation is used to extract three common divisors. KMO and the Bartlett’s test of sphericity are shown in Table 2.

**Table 2:** Patient participation and KMO’s and Bartlett’s test.

KMO sample measurement		0.83
Bartlett’s test	Chi-square estimation	1447.14
	Degree of freedom	36
	Significance probability	0.00


It is shown in Table 2 that KMO value is 0.83, which is more than 0.8, and it indicates that factor analysis is available. The Chi-square value of Bartlett’s Test of Sphericity is 1447.14, and its significance probability is 0.00 (<0.01), which is suitable for factor analysis.

The total variance explained rate of three extracted factors is 68.82%, which indicates that three extracted factors reflect most of the information of the original item; see Table 3.

**Table 3:** Characteristic values of the extracted common factors for patient participation.

Common factor	Initial characteristic value	Explained variation after factor rotation%	Accumulatively explained variation after factor rotation%
1	3.84	42.64%	42.64%
2	1.38	15.34%	57.98%
3	0.98	10.84%	68.82%


The loading matrices and communality of three extracted factors are shown in Table 4.

**Table 4:** Factor loading matrices for patient participation.

Renamed main factor	Measuring items	Factor load			Communality
		1	3	3	
Information search	I have generally understood the information concerning the hospital or the doctor that I will visit before seeing a doctor.	0.78	0.18	0.14	0.66
	I have generally understood my own symptoms and how I will be treated before visiting the doctor	0.87	0.15	0.50	0.78
	I have searched for the relevant information, and I know what I should assist my doctor to do before medical diagnosis or treatment	0.80	0.20	0.16	0.69
Responsible behavior	I will try my best to describe my symptoms to the doctor during medical diagnosis and treatment	0.20	0.74	0.24	0.65
	I will complete medically necessary procedures as required in the process of medical diagnosis and treatment	0.20	0.84	0.10	0.76
	I will cooperate fully with the doctor and follow the requirements the doctor make	0.16	0.80	0.25	0.73
Interpersonal interaction	I will offer useful suggestions to improve the quality of the medical services	0.40	0.50	0.63	0.57
	I will take the initiative to communicate with my doctor during diagnosis and treatment	0.07	0.35	0.73	0.65
	I will do what I can do to help other patients if necessary	0.03	0.16	0.82	0.70


According to the factor loading matrices for the patient participation, the communality of all variables are more than 0.5, which indicates that three factors can at least explain 50% of the item variance; all the load factors are more than 0.5, which indicates that there is high relevance between the factors and the measuring items, and every measuring item has high load on its corresponding factor. It further indicates that 3D patient participation model is an ideal analysis model.

### Verification of Assumption

**FIGURE 2:**
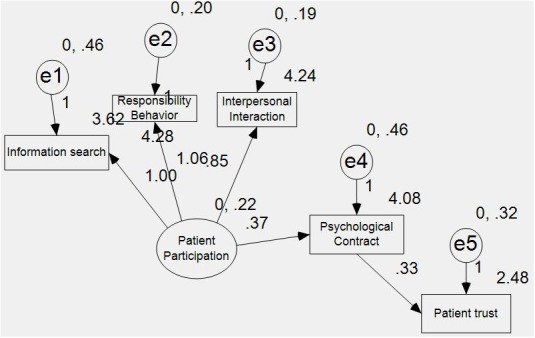
Standardized path figure between patient participation, patient’s psychological contract and patient trust.

AMOS6.0 statistical analysis software together with the survey data is used to test the conceptual model, and Figure 2 is a standardized path figure.

It can be seen that all path coefficients reach the statistical significance. In addition, the Chi-square value is 27.69, and the degree of freedom is 5, and P value is 0.00, reaching the statistical significance. It thus can be argued that the fitting degree of the conceptual model is good. According to the fitting indices, CFI = 0.94, NFI = 0.93, and IF = 0.94, all of which indicate that the fitting degree of the model is perfect. This verifies the theoretical assumption proposed in this paper. First, patient participation is an antecedent variable for the patient’s psychological contract and patient trust. Second, the patient’s psychological contract is a mediator variable between the patient participation and the patient’s trust in doctor.

In order to further judge whether there is a complete mediation, partial mediation or non-mediation relationship between the patient’s psychological contract, the patient participation and patient trust, this paper tests the fitting degree of the model without mediation and the model with partial mediation.

The fit indices without mediation are: CFI = 0.80, NFI = 0.79, and IFI = 0.80. In terms of the judgment index, the fitting degree of the model without mediation is not higher than the model with complete mediation, and we thus choose the model with complete mediation. However, the Chi-square value of the model with partial mediation is 2.63, and its degree of freedom is 4, and *P*-value is 0.62, without reaching the statistical significance. Therefore, we give up the model with partial mediation.

## Discussion and Conclusion

This study reaches the following three conclusions: first, patient participation is an antecedent variable for the patient’s psychological contract and patient trust; second, the patient’s psychological contract is a mediator variable between the patient participation and the patient’s trust in doctor; third, patient participation can be categorized as three dimensions: information search, responsible behavior, and interpersonal relationship. Based on our analysis above, three implications can be highlighted.

First, focusing and encouraging patient participation. Being influenced by the traditional medical service mode, the doctor makes insufficient and incomplete disclosure of medical information to patients and the doctor is just informed of the diagnosis scheme and therapeutic results, and the patient would not be allowed to participate in the process of medical decision-making such as the judgment of the symptoms and pathological analysis. It is neither good for establishing the patient’s psychological contract, nor good for cultivating the patient’s trust in the doctor. Thus, we should try our best to promote and popularize the shared medical decision making, thereby making the patient fully participate in the decision-making. The shared decision-making indicates that the doctor and patient are the equal participants, and both the doctor and the patient are entitled to participate in treatment decision making; the shared decision-making requires the physician to inform the patients of all relevant information concerning the treatment, and requires the patient to make medical decision under the guidance of the doctor after effective communication between them.

The patient participation includes three components: information search, responsible behavior and interpersonal interaction. The responsible behavior that is indispensable for the medical service, refers to the practice that must be completed by the patient while receiving the diagnosis and treatment; information search refers to the behavior that the patient actively learns his/her symptoms and information about the hospital and the doctor involved by different ways before receiving the diagnosis and treatment; The interpersonal interaction refers to emotional and informational exchanges between the doctor and patient before receiving diagnosis and treatment.

The responsible behavior is characterized as the fundamental participation level, and the information search and interpersonal interaction are characterized as the active and higher participation level of the patient.

If the patient has known the treatment process in advance and understood some basic medical knowledge, the cognitive conflict brought by the information asymmetry will be reduced. If the patient has fully known his/her rights and obligations, the patient’s role as a passive recipient of care will be changed. In addition, the patient can also strengthen the relationship between doctors and patients and improve the quality of the medical service by developing a personal friendship with the doctor.

Second, focusing on stabilizing and strengthening the patient’s psychological contract. The patient’s psychological contract is an inherently subjective practice, which is a combination of the emotional fitness and commitment fitness. The patient’s psychological contract can be characterized as the complexity and uncertainty of the content, the urgency of situation, the anxiety of the patient as well as the priceless quality of the subject matter (Yang and Su, 2015). The approaches to stabilize and strengthen the patient’s psychological contract are as follows: (1) improving the moral character of both the doctor and the patient; (2) strengthening the positive feedback between “commitment—action—trust”, in order to make the psychological contract between doctors and patients clear and stable and reduce the violation of the psychological contract between doctors and patients; (3) a sensible combination of reward and punishment as driving force of rebuilding mutual trust between doctors and patients.

Third, achieving the synergic improvement of the interpersonal trust and general trust between doctors and patients. Patient trust can be categorized as the general trust in the medical system and the interpersonal trust in the treating physician. When the medical care starts, the interpersonal trust between doctors and patients is established. With the establishment of the interpersonal trust, the patient’s original belief in the medical system may be changed. Thus, the mutual influence between the general trust and the interpersonal trust will run through the whole process of establishing and maintaining the relationship between doctors and patients.

## Author Contributions

TY wrote the manuscript and designed the questionaire. YW formulated the framework of the manuscript.

## Conflict of Interest Statement

The authors declare that the research was conducted in the absence of any commercial or financial relationships that could be construed as a potential conflict of interest.

## References

[B1] Blancero D. Ellram L. (1997). Strategic supplier partnering: a psychological contract perspective. *Int. J. Phys. Distrib. Logistics Manag.* 27 616–629. 10.1108/09600039710188684

[B2] Chen K. M. Zhang X. J. Zheng Y. N. (2013a). A study on the establishment of the harmonious relationship between doctors and patients from the perspective of psychological contract. *J. Gannan Med. Univ.* 5 23–27.

[B3] Chen K. M. Zhang X. J. Zheng Y. N. (2013b). The analysis of the connotation, elements and function of the patient’s psychological contract. *Econ. Res. Guide* 24 292–294.

[B4] Dong E. H. Bao Y. (2012). The reliability and validity of the revised Chinese version of the wake forest physician trust scale. *Chin. Men. Health J.* 26 171–175.

[B5] Geng X. F. (2008). *A Study on the Influence of the Measuring Dimension of Client Participation, and Driving Factors on Client Satisfaction — Based on Sample Survey of Hangzhou Medical Service Industry.* Doctoral dissertation, Zhejiang University, Hangzhou.

[B6] Guieford J. P. (1965). *Fundamental Statistics in Psychology and Education.* New York, NY: McGraw-Hill.

[B7] Li L. (2010). Regaining the trust is one of the standards that the reform of the public hospitals makes success. *Chin. Med. Insur.* 5 34–34.

[B8] Li W. (2005). Red envelope, trust and institution. *J. Sun Yat Sen Univ.* 45 110–117.

[B9] Luo H. (2006). An empirical analysis of psychological contract mechanism of customer loyalty. *Manag. Rev.* 18 57–62.

[B10] Luo H. C. Fan X. C. (2005). The relationship marketing mechanism based on the psychological contract: the empirical study on the service industry. *Nankai Bus. Rev.* 6 48–55.

[B11] Mainous A. G. Kerse N. Brock C. D. Hughes K. Pruitt C. (2003). Doctor developing patient trust: perspectives from the United States and New Zealand. *N. Z. Fam. Physician* 30 336–341. 24972064

[B12] Nunnally J. (1978). *Psychometric Theory.* New York, NY: McGraw-Hill.

[B13] Yang Y. (2007). *Trust Being the Inherent Value of the Relationship Between Doctors and Patients.* Doctoral dissertation, Dalian Medical University, Dalian.

[B14] Yang T. W. Su Y. G. (2015). Discussion on the influence of “refusing red envelope” on the psychological contract between doctors and patients— from the patient’s point of view. *Shandong Soc. Sci.* 10 189–192.

[B15] Yu J. L. Li Y. Ni J. (2011). The empirical study on the repeat purchase intention by the network consumers based on the psychological contract. *Theory Pract. Finance Econ.* 32 96–100.

[B16] Zheng D. X. (2010a). The ethical dimension and legal support for the harmonious relationship between doctors and patients. *Chin. Med. Ethics* 23:41

[B17] Zheng D. X. (2010b). The lack and rebuilding of trust between doctors and patients in sociological context. *Med. Society* 23 14–16.

[B18] Zou S. (2010). The establishment of harmonious relationship between doctors and patients from the perspective of the psychological contract. *Chin. Med. Ethics* 23 117–119.

